# Host Community Traits Driving Crimean‐Congo Hemorrhagic Fever Virus Maintenance in Iberian Ecosystems

**DOI:** 10.1155/tbed/1152849

**Published:** 2026-03-03

**Authors:** Patrícia Xavier, Alberto Perelló, Víctor Luque-Castro, David Relimpio, Patricia Barroso, Virgílio Almeida, José de la Fuente, Ana Balseiro, Francisco Ruiz-Fons, Christian Gortázar

**Affiliations:** ^1^ Health and Biotechnology (SaBio) Group, Instituto De Investigación En Recursos Cinegéticos (IREC) CSIC-UCLM-JCCM, Ciudad Real, Spain; ^2^ Department of Animal Health, Faculty of Veterinary Medicine, University of León, León, Spain, unileon.es; ^3^ Centre for Interdisciplinary Research in Animal Health (CIISA), Faculty of Veterinary Medicine, University of Lisbon, Lisbon, Portugal, ulisboa.pt; ^4^ Department of Veterinary Pathobiology, Center for Veterinary Health Sciences, Oklahoma State University, Stillwater, Oklahoma, USA, okstate.edu; ^5^ Instituto de Ganadería de Montaña (IGM, CSIC-ULE), León, Spain; ^6^ Biomedical Research Networking Center for Infectious Diseases (CIBERINFEC), National Health Institute Carlos III, Madrid, Spain

**Keywords:** biodiversity, camera trap, Crimean-Congo hemorrhagic fever virus, land-use change, multihost diseases, one health, tick-borne diseases

## Abstract

Crimean‐Congo hemorrhagic fever (CCHF) is a tick‐borne zoonosis of significant public health concern, maintained in complex multihost systems shaped by ecological, climatic, and anthropogenic drivers. In the Iberian Peninsula, changing land‐use patterns and biodiversity loss may be reshaping host communities in ways that alter local transmission dynamics. We conducted a landscape‐scale study across 18 sites, integrating serological surveillance of wild ungulates (*n* = 1461; 69.4% wild boar, 30.6% red deer) with camera‐trap monitoring of mammalian communities, land cover analysis, and climatic data. To capture ecological drivers at different scales, we fitted two generalized linear mixed models (GLMMs): one including all sites to identify broad landscape‐level predictors of exposure, and another restricted to endemic sites to evaluate fine‐scale dynamics within established transmission foci. Overall, 44.5% of sampled individuals tested positive for CCHF virus (CCHFV) antibodies, with site‐level seroprevalence ranging from 1.5% to 81.4%. Across all sites, seroprevalence was positively associated with red deer abundance, underscoring the potential role of red deer as key amplifying host, forest cover, and precipitation seasonality, while small ruminant presence was linked to reduced exposure risk. Within endemic areas, higher mammalian diversity and greater lagomorph abundance were negatively associated with exposure, whereas warmer temperatures promoted circulation. This pattern suggests that more balanced host communities can reduce the efficiency of pathogen transmission. Overall, this study highlights how community structure and environmental change jointly shape CCHFV ecology. The context‐dependent nature of ecological drivers support integrated One Health strategies that conserve biodiversity, promote mixed grazing systems, and regulate wild ungulate populations to reduce CCHFV circulation in Mediterranean ecosystems undergoing socioecological transformation.

## 1. Introduction

Early epidemiological research on zoonotic infectious diseases focused on understanding the relationship between pathogens and host species exhibiting symptoms, typically humans and domestic animals. Thus, prevention and eradication strategies were based on a single‐host perspective, failing to consider the potential involvement of other organisms in pathogen persistence and transmission [1]. While several human and animal infectious diseases share these simple epidemiological dynamics, scientific evidence shows that most infectious agents circulate in far more complex transmission cycles involving multiple animal species [2]. Indeed, multihost pathogens such as the Crimean‐Congo hemorrhagic fever virus (CCHFV) (*Orthonairovirus haemorrhagiae*) are currently responsible for the largest proportion of emerging and re‐emerging infectious diseases [3].

Crimean‐Congo hemorrhagic fever (CCHF) is one of the most widespread tick‐borne viral diseases globally and an increasing public health concern [4]. Since 2013, an increased number of autochthonous human cases were reported in the Iberian Peninsula—20 in Spain and one in Portugal—of which seven were fatal[5, 6]. The recent emergence of the disease in Iberia appears to be driven by ecological, climatic, and anthropogenic changes affecting the CCHF epidemiological system [7, 8]. Nevertheless, CCHFV has circulated in the region for decades, maintained in a silent tick–vertebrate–tick enzootic cycle [9]. Multiple independent introduction routes, including migratory birds and livestock movements, likely contributed to the genetic heterogeneity currently observed in Iberia. Human cases have involved Genotypes III and V, as well as Genotype IV (Africa 4 clade), a novel reassortant strain first detected in 2018 [10, 11]. Tick‐based studies similarly reveal a diverse viral landscape, with Genotypes I, III, IV, and V being detected [12–15]. Recently, CCHFV was also reported for the first time in France in ticks, with sequences clustering within Genotype III [16], one of the predominant genotypes circulating in the peninsula.

In Iberia, serological surveys against CCHFV antibodies reveal high exposure in wild ungulates, particularly red deer (*Cervus elaphus* Linnaeus, 1758) and wild boar (*Sus scrofa* Linnaeus, 1758), with seroprevalence up to 88% in southwestern regions [17, 18]. Domestic ungulates, by contrast, display much lower exposure levels [19]. Small ruminants show only around 3% seropositivity [20, 21], while a recent study found a higher prevalence (around 38%) in cattle from northeastern Portugal [22]. Lagomorphs have consistently tested seronegative, even in areas with high viral circulation [23, 24]. However, recent evidence from Portugal identified isolated cases of seropositivity (0.6%) in wild rabbits [25].

*Hyalomma marginatum Koch*, *1844* and *Hyalomma lusitanicum* Koch, 1844 ticks are the primary vectors and reservoirs of CCHFV in Iberia, playing a central role in its maintenance [14, 26]. These ticks can acquire the virus at any life stage through feeding on viremic hosts or co‐feeding and maintain it via transstadial and transovarial transmission [27]. Both species exhibit diatropic feeding behavior—parasitizing different host species during immature and adult stages [28]—and act as host generalists, adapting to local host availability, and facilitating virus circulation within multihost communities [29–31]. The main hosts of *H. marginatum* immature ticks are small mammals, particularly lagomorphs, and ground‐feeding birds, while adults prefer large domestic ungulates, but also infest wild hosts depending on local host community composition [28]. *Hyalomma lusitanicum* larvae and nymphs typically parasitize European rabbits (*Oryctolagus cuniculus* (Linnaeus, 1758)), whereas adults favor wild ungulates like red deer and wild boar [32]. However, immature ticks are also known to parasitize these larger hosts [33, 34].

Hosts play a critical role in tick‐borne diseases by supporting vector populations and contributing to pathogen circulation [8]. In this way, host identity and host diversity can shape transmission dynamics [35]. For example, some species such as the white‐footed deermouse (*Peromyscus leucopus* (Rafinesque, 1818)) for *Borrelia burgdorferi* and the American robin (*Turdus migratorius* Linnaeus, 1766) for West Nile virus in the USA are key amplifying hosts, contributing abundantly for pathogen spread [36, 37]. The same is true for CCHFV, where not all animal species contribute equally to its maintenance [38]. Most birds are refractory to infection and act as non‐competent hosts, while mammals can replicate the virus and transmit it to naive feeding ticks, thus, acting as competent hosts [39]. All susceptible species experience only a brief period of viremia, limiting the window for horizontal systemic transmission [30]. However, competent vertebrates with high tick burdens, such as large mammals, can infect many feeding ticks simultaneously contributing disproportionately to the persistence of viral foci, while less competent hosts play only a minor role [38, 40]. The dominance of such species in low‐diversity communities may increase pathogen circulation in the environment and represent a public health threat [41]. Conversely, more diverse communities with a higher proportion of less competent or non‐competent species may reduce viral replication [42]. This dilution effect was first documented in Lyme disease [43]. Nonetheless, this buffering is context dependent: in some systems, added diversity may include more competent hosts or promote vector proliferation, leading instead to amplification [44].

Although the relationship between biodiversity and disease risk has been explored in other vector‐borne disease episystems [45], its influence on the CCHF epidemiology is underexplored. Moreover, despite evidence of varying exposure levels among wild and domestic species, the broader ecological context—particularly how multihost community composition shapes transmission dynamics—remains poorly understood. To address this knowledge gap, we conducted a landscape‐scale study across ecologically diverse sites in the Iberian Peninsula. We aimed to clarify the epidemiological roles of different mammal hosts in sustaining CCHFV, assess the role of mammalian biodiversity in virus transmission, and identify key biotic and abiotic predictors of exposure risk, including host community metrics, habitat characteristics, and climatic variables.

## 2. Materials and Methods

### 2.1. Study Design

We conducted a retrospective cross‐sectional study at 18 integrated wildlife monitoring (IWM) points across distinct bioregions of the Iberian Peninsula. These sites were strategically selected to capture regional variation in wildlife management, habitat, and climate [46] (Supporting Information 1: Figure S1 and Table S1). At each IWM point, active disease surveillance and population monitoring were integrated to assess the role of wildlife in CCHFV dynamics. Camera traps (CTs) were deployed to monitor wildlife host communities, with a 5‐km buffer zone defined around the centroid of each CT network. This buffer served to standardize the collection of both ecological and serological data. Wild boar and red deer were selected as sentinel species due to their abundance, broad distribution, role as *Hyalomma* spp. tick hosts, and suitability for serological surveillance [17, 18, 47]. Serum samples were collected within this buffer and analyzed to determine both the individual serological status and the overall seroprevalence of CCHFV antibodies at each study site. Spatial data processing was performed using QGIS (v3.36.3).

### 2.2. Sample Collection and Serological Analysis

A total of 1461 blood samples were collected between 2018 and 2024 from animals distributed in 18 study sites in the Iberian Peninsula. Samples were taken from two species, wild boar (69.4%) and red deer (30.6%). The number of animals sampled per point ranged from 16 to 249 (Figure 1b). Red deer were sampled at seven sites, as they were either absent or present in very low numbers at the other study sites, while wild boar were sampled at all 18 points (Supporting Information 1: Figure S1 and Table S1). Blood was drawn from the endocranial venous sinus of hunted animals [48]. The blood samples were centrifuged at 4000 × *g* for 5 min to extract serum, which was then stored at −20°C until testing. Serological analysis was conducted using the IDScreen CCHF Double Antigen Multispecies ELISA kit (IDVet, Grabels, France), which detects both IgG and IgM antibodies against CCHFV, following the manufacturer’s instructions. The assay has a reported sensitivity of 98.9% and specificity of 100%. According to the manufacturer, the ELISA shows no cross‐reactivity with other orthonairoviruses such as Hazara virus, Dugbe virus, and Nairobi sheep disease virus; however, cross‐reactivity with Aigai virus (formerly classified as CCHFV genotype VI) has not been evaluated [49, 50].

Figure 1Seroprevalence of Crimean‐Congo hemorrhagic fever virus (CCHFV) antibodies and sample distribution by study site. (a) Estimated seroprevalence (%) of CCHFV antibodies across study sites. (b) Counts of seropositive and seronegative samples by species and study site. Subfigure (b1) shows results for red deer, and Subfigure (b2) for wild boar.

**Figure:**
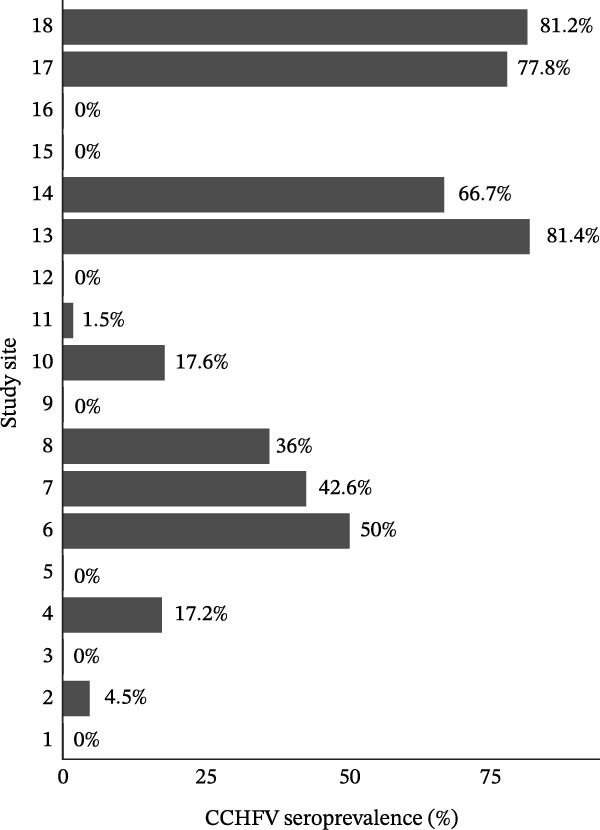
(a)

**Figure:**
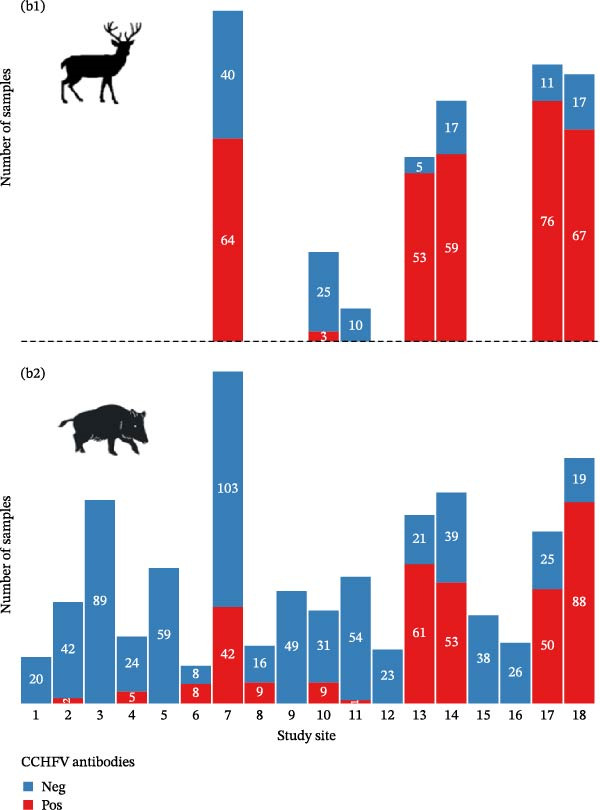
(b)

### 2.3. Multihost Community Monitoring and Characterization

From 2020, an average of 20 unbaited CTs (Browning Strike Force HD ProX, Browning Arms Company, Morgan, Utah, USA) were deployed annually at each IWM site. Cameras were arranged in a random grid with 1 km spacing and operated continuously for one to 2 months. Each unit was positioned in a fixed location, facing north, 50 cm above ground, and aligned parallel to the terrain slope. Traps operated 24 hr, taking eight consecutive images per trigger event, with a 1 s delay between successive activations. Individuals photographed over 120 s apart were treated as independent encounters. After retrieval, long‐term nonoperational CTs were excluded from analysis. The trapping effort was standardized at 450 camera‐days, representing the total number of days that the cameras operated collectively in the field. This approach ensured a comparable sampling effort across the different study sites [51].

Based on these data, we determined the relative abundance index (RAI), namely, trapping rate (TR), and the relative occupancy index (ROI) for 27 mammal species [52–54]. These indices were used to derive relative weight (RW) for each species, considering both capture frequency and spatial distribution. Taxa comprising morphologically similar species that are difficult to distinguish in camera‐trap images were grouped together (e.g., lagomorphs). The relative interaction index (RII) was calculated for species pairs relevant to CCHFV epidemiology. It was based on indirect interactions, defined as detections of different species by the same camera within 24 h [55]. The Shannon diversity index (*H*′) was also estimated using the *vegan* R package (v2.6‐4), based on species detection data obtained from camera trap records. As it includes only captured species, this metric is termed “apparent diversity index” [56]. All indices are defined and described in Supporting Information 2.

### 2.4. Environmental Predictors

We selected a combination of host and environmental predictors (Table 1) to model the odds of CCHFV exposure in the wild ungulates studied. Environmental variables were extracted within the 5‐km buffer around each study site using QGIS (v3.36.3), as this scale is representative of the surrounding landscape context.

**Table 1:** List of explanatory predictors considered relevant to Crimean‐Congo hemorrhagic fever disease dynamics.

Factor	Predictor	Meaning (unit of measure)	Average (range)
Host population	TR_Wb	Trapping rate for wild boar	0.357 (0.049, 0.826)
	TR_Rd	Trapping rate for red deer	0.706 (0.000, 2.846)
	TR_Lag	Trapping rate for lagomorphs	0.147 (0.000, 2.020)
	TR_Cattle	Trapping rate for cattle	0.022 (0.000, 0.152)
	TR_SR	Trapping rate for small ruminants	0.010 (0.000, 0.088)
	RII_WbRd	Relative interaction index between wild boar and red deer	2.942 (0.000, 13.314)
	RII_WildDome	Relative interaction index between wild hosts (wb and rd) and domestic hosts (cattle and small ruminants)	0.022 (0.000, 0.202)
	Shannon_Mammals	Shannon diversity index of mammals	1.335 (0.11, 2.292)
Habitat	Shrub	Shrub cover (%)	32.943 (5.886, 70.480)
	Grass	Grass cover (%)	12.283 (0.000, 73.109)
	Forest	Forest cover (%)	32.093 (2.341, 63.944)
Bioclimatic	LST	Mean annual land surface temperature (°C)	23.536 (16.071, 27.294)
	NDVI	Mean annual normalized difference vegetation index	0.515 (0.333, 0.715)
	PSeas	Precipitation seasonality (%)	49.325 (21.488, 70.228)

Forest and shrub cover were included as environmental predictors of habitat, based on prior studies linking these land types to *Hyalomma* spp. abundance [28]. Land use data was sourced from the CORINE Land Use/Land database, with a spatial resolution of 100 m [57]. Three bioclimatic variables were also selected: land surface temperature (LST), normalized difference vegetation index (NDVI), and precipitation seasonality (PSeas). LST data were obtained from MODIS, with an 8‐day temporal resolution and 1 km spatial resolution (2000–2024) [58]. NDVI data, also from MODIS, were available every 16 days at 500 m resolution (2015–2024) [59]. PSeas data were sourced from the WorldClim 2 project, representing average values from 1970 to 2000 at 1 km resolution [60]. This variable is calculated as the coefficient of variation of monthly precipitation and, therefore, reflects the relative degree of intra‐annual rainfall variability. These variables were selected due to their relevance in shaping tick ecology and virus transmission (Supporting Information 3: Table S2).

### 2.5. Statistical Analyses

Host community composition at each site was quantified by calculating the RW of each detected species. Red deer, wild boar, lagomorphs, cattle, and small ruminants were analyzed individually due to their known or potential role in CCHF epidemiology [27], while all other species were grouped into a general category. For comparative analyses, sites were classified into null (0%, *n* = 7) and high prevalence (≥50%, *n* = 5). The 50% threshold was chosen to represent the upper tail of the prevalence distribution (median = 10.9%, IQR = 0.0%–48.1%), thereby distinguishing sites with intense circulation from those with absent or lower exposure. Differences between groups were tested with Wilcoxon rank–sum.

To predict the odds of exposure to CCHFV, generalized linear mixed models (GLMMs) [CCHFV antibodies ~Host predictors + environmental predictors + (1 | study site)] were employed using the *lme4* R package (v1.1‐33) [61]. Two datasets were analyzed: the first included all 18 sites (*n* = 1461 individuals), and the second was restricted to the 11 sites with evidence of previous exposure to the virus (*n* = 1157 individuals). In both cases, the response variable was the individual‐level serological status (presence/absence of antibodies), fitted with a binomial distribution and a logit link. This approach enabled the analysis of a larger data set. The study site was included as a random effect. Fixed effects included the TR of known *H. lusitanicum* and *H. marginatum* hosts (wild boar, red deer, lagomorphs, cattle, and small ruminants), RIIs between wild boar and red deer and between wild and domestic ungulate hosts, as well as the Shannon diversity index. Environmental predictors (shrub, grass, and forest cover; LST; NDVI; PSeas) were also included. To address multicollinearity, we examined pairwise correlations and VIF values for all covariates. We retained the predictor considered most ecologically meaningful for CCHFV epidemiology when two variables were highly correlated, ensuring that all variables included in the models had a VIF <3, a conservative threshold commonly applied in ecological studies [62]. Model selection was based on the corrected Akaike’s Information Criterion (AIC). The best‐fitting models had AIC = 1282.36 for the analysis across all 18 sites and AIC = 1248.65 for the analysis restricted to the sites with positive individuals. Models were built manually by generating all possible combinations of predictor variables. Post‐selection diagnostics were conducted to verify residual normality and the absence of systematic patterns. Model performance was evaluated using the area under curve (AUC). Models’ results were reported as odds ratios (ORs) with 95% confidence intervals (CIs). R’s *effects* package [63] was used to visualize the relationship between each predictor and the response variable. All analysis in this study were performed using R software, version 4.4.1.

## 3. Results

### 3.1. Seroprevalence of CCHFV Antibodies

Serological analyses revealed that 650 individuals had CCHFV antibodies (44.5%, CI: 41.9–47.1). Positive samples were found in 11 study sites. At the site level, overall seropositivity ranged from 1.5% to 81.4% (43.3% ± 30.5 Figure 1a). While species‐specific seropositivity varied from 1.8% to 82.2% (40.2% ± 27.8) in wild boars and from 10.7% to 91.4% (58.3% ± 37.5) in red deer. The proportion of positive individuals was higher in southwestern Iberia, where all populations with a prevalence ≥50% (5/18; 27.8%) were identified. In contrast, almost all Northern Iberian sites had no positive samples, except for study Site 2, where a prevalence of 4.5% (2/44) was detected.

### 3.2. Effect of Host Community Composition and Diversity

Analysis of host community composition revealed clear differences between high (≥50%) and null (0%) CCHFV seroprevalence sites. Regarding wild hosts, red deer exhibited a significantly higher RW at high seroprevalence sites, ranging from 55.6% to 84.2%, compared to seronegative sites, where RW ranged from 0.0% to 21.4% (*W* = 35, *p* = 0.004). In contrast, wild boar RW did not differ significantly between high prevalence negative sites (*W* = 9, *p* = 0.193), and lagomorphs also showed no significant difference (*W* = 14, *p* = 0.626; Figure 2). Domestic hosts (cattle and small ruminants) were not detected at high seroprevalence sites (RW = 0%). At seronegative sites, their presence was limited: cattle had a RW of 0.0%–5.4%, and small ruminants ranged from 0.02% to 3.3%. A significant difference in RW between the two site categories was found for small ruminants (*W* = 0, *p* = 0.004), but not for cattle (*W* = 10, *p* = 0.135; Figure 2). Finally, the RW of all other species not classified as primary hosts of *Hyalomma* ticks was significantly higher at seronegative sites (14.5%–79.4%) than at high seroprevalence sites (5.1%–21.7%; *W* = 2, *p* = 0.014; Figure 2). Summary statistics for all species groups are provided in Supporting Information 4: Table S3.

Figure 2Quantitative composition of the Crimean‐Congo hemorrhagic fever virus maintenance host community in study points with a high (a) and null (b) prevalence of antibodies to the virus. Species less involved in the epidemiological cycle of this disease are included under “Other species.” The proportion of each species represents their relative weight (RW) calculated from two relative abundance indices: the trapping rate (TR) and the relative occupancy index (ROI) of each species at each point. The relative weight of each species at each study point is described in Supporting Information 5: Table S4.

**Figure:**
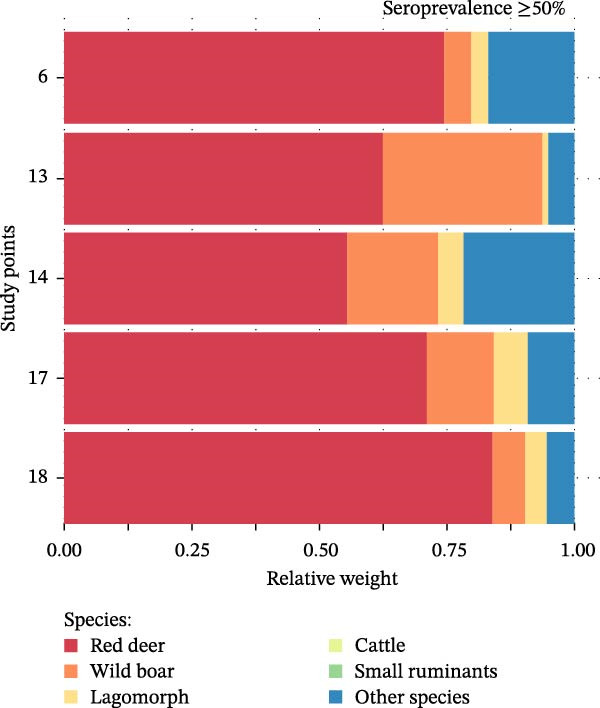
(a)

**Figure:**
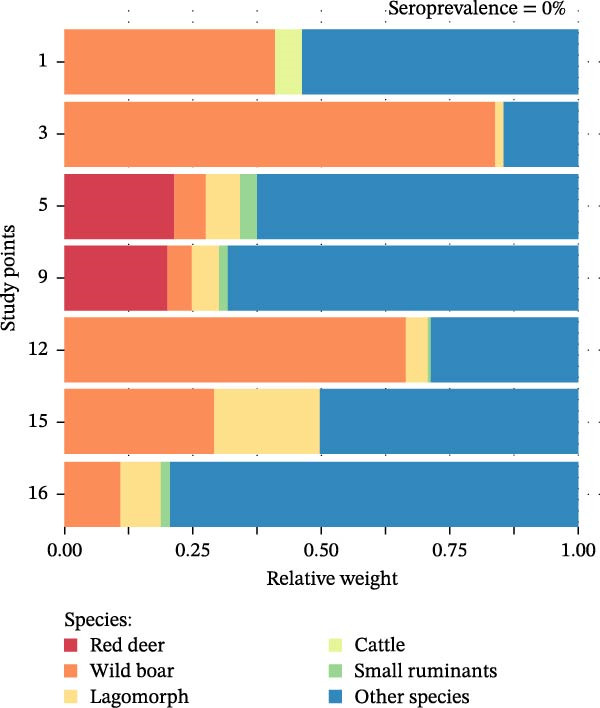
(b)

### 3.3. Ecological Determinants of CCHFV Exposure in Complex Episystems

The best‐fitting model for the full dataset (18 sites, *n* = 1461) included several explanatory variables: TR of red deer and small ruminants, RII of wild (red deer and wild boars) and domestic hosts (cattle and small ruminants), PSeas, and proportion of forest cover (Table 2 and Supporting Information 6: Figures S2 and S3). Model residuals were adequately dispersed and normally distributed, and the model demonstrated strong discriminatory capacity (AUC = 0.87). Regression analysis identified a statistically significant relationship between all these variables, except for the RII between wild and domestic hosts, and the likelihood of exposure to CCHFV (Table 2). The odds of exposure to the virus tended to increase with the abundance of red deer. In contrast, a higher TR of small ruminants was associated with a reduced risk of exposure. Among environmental variables, PSeas showed a strong positive association, with an eightfold increase in odds associated with higher seasonality. Similarly, greater forest cover was linked to an elevated risk, with each unit increase in forest percentage associated with a 3.93‐fold rise in the odds of exposure.

**Table 2:** Results of the best‐fit generalized linear mixed model (GLMM) for Crimean‐Congo hemorrhagic fever virus antibodies presence, expressed as odds ratios, based on all 18 study sites.

Predictor	Est.	2.5%	97.5%	Std. error	*Z* value	*p*‐Value
Intercept	0.228	0.086	0.602	1.641	‐2.986	0.00283**^∗∗^**
TR_Rd	2.581	1.171	5.686	1.496	2.353	0.01865**^∗^**
TR_SR	0.442	0.199	0.978	1.501	‐2.014	0.04404**^∗^**
RII_WildDome	1.656	0.990	2.768	1.300	1.924	0.05441.
PSeas	8.663	2.118	35.427	2.051	3.005	0.00266**^∗∗^**
Forest	3.935	1.010	14.078	1.916	2.106	0.03518**^∗^**

*Note:* It includes estimates of odds ratios (Est.), 95% confidence intervals (2.5% and 97.5%), Std. error, *Z* values, and *p*‐values indicating the statistical significance of each predictor. Significance codes: 0 ‘ ^∗∗∗^’ 0.001 ‘ ^∗∗^’ 0.01 ‘ ^∗^’ 0.05 ‘.’ 0.1 ‘ ’ 1. Predictor codes: TR_Rd = trapping rate for red deer; TR_SR = trapping rate for small ruminants; RII_WildDome = relative interaction index between wild hosts (wild boar and red deer) and domestic hosts (cattle and small ruminants); PSeas = precipitation seasonality (%); Forest = forest cover (%).

Abbreviation: Std. error, standard error.

When the analysis was restricted to the 11 sites with evidence of previous exposure to the virus (*n* = 1157), the selected model included TR of lagomorphs and small ruminants, mammalian diversity, (Shannon index), LST, and grass cover (Table 3 and Supporting Information 6: Figures S2 and S3). This model also showed adequate fit and good discriminatory performance (AUC = 0.79). Regression analysis indicated that exposure risk decreased with higher TR of lagomorphs and with increasing mammalian diversity, whereas warmer LST was positively associated with exposure. Grass cover and small ruminant abundance were not significant predictors.

**Table 3:** Results of the best‐fit generalized linear mixed model (GLMM) for Crimean‐Congo hemorrhagic fever virus antibodies presence, expressed as odds ratios, based on the 11 sites with confirmed viral circulation.

Predictor	Est.	2,5%	97,5%	Std. error	*Z* value	*p*‐Value
Intercept	1.232	0.980	1.548	0.117	1.789	0.07369.
TR_Lag	0.640	0.544	0.753	0.083	‐5.372	0.7805e−07 ^∗∗∗^
TR_SR	1.043	0.773	1.407	0.153	0.273	0.7851
Shannon_Mammals	0.303	0.219	0.417	0.164	‐7.288	0.3152e−12 ^∗∗∗^
LST	1.660	1.284	2.144	0.131	3.870	0.1088e−03 ^∗∗∗^
Grass	0.980	0.810	1.183	0.097	‐0.220	0.8262

*Note:* It includes estimates of odds ratios (Est.), 95% confidence intervals (2.5% and 97.5%), Std. error, *Z* values, and *p*‐values indicating the statistical significance of each predictor. Significance codes: 0 ‘ ^∗∗∗^’ 0.001 ‘ ^∗∗^’ 0.01 ‘ ^∗^’ 0.05 ‘.’ 0.1 ‘ ’ 1. Predictor codes: TR_Lag = trapping rate for lagomorphs; TR_SR = trapping rate for small ruminants; Shannon_Mammals = Shannon diversity index of mammals; LST = mean annual land surface temperature (°C); Grass = grass cover (%).

Abbreviation: Std. error, standard error.

## 4. Discussion

The integration of active surveillance with animal population monitoring provides new insights into how host community composition and diversity modulate CCHFV transmission [64]. At the study site level, we found that areas with high seroprevalence were dominated by red deer, reflecting a simplified community structure with limited representation of other host species. In contrast, seronegative sites exhibited greater species diversity, with a significantly higher proportion of non‐primary CCHFV hosts (Figure 2). These findings highlight the potential role of red deer as a key amplifying host and suggest a protective role of balanced host communities against the virus.

In the Iberian Peninsula, CCHFV circulation is primarily maintained through an enzootic sylvatic cycle involving *H. lusitanicum* ticks and wild ungulates [14]. Previous studies identified red deer as a valuable model for understanding the ecological drivers of CCHF dynamics [17] largely due to their role as the main host of *H. lusitanicum*, one of the principal vectors of the virus in this region [32]. The strong positive association we observed between red deer dominance and CCHFV seroprevalence in our study further supports the role of this species as a putative amplification host. This likely reflects the substantially higher tick burden these individuals carry compared to other host species [65]. This close tick‐host interaction can promote CCHFV amplification through multiple complementary mechanisms: (1) enabling simultaneous infection of multiple ticks during viremic periods, (2) co‐feeding, as infected and uninfected tick clusters in specific body regions, facilitating non‐systemic transmission [29], and (3) indirectly, by sustaining large *Hyalomma* spp. populations, which represent the primary reservoir of the virus [13]

Interestingly, all deer‐dominated communities were in fenced hunting estates in South‐Central Spain, where wild ungulates are commonly overabundant. These areas are typically characterized by high densities and spatial aggregation of large game species such as red deer and wild boar [66, 67]. The proliferation of these ecological important hosts on CCHFV episystem [17, 18], together with the management practices that promote it (e.g., alteration of habitat structure), may have disrupted the ecological balance of those ecosystems and led to the decline and disappearance of more vulnerable species, including nonprimary CCHFV hosts, which could contribute to viral dilution [68, 69].

Wild boar was also recognized as a reliable sentinel species for CCHFV exposure, exhibiting high seroprevalence rates both in our study and in previous research [18]. However, our findings suggested this species may play a less central role in sustaining local CCHFV transmission compared to red deer. This difference may be explained by the higher *H. lusitanicum* burden typically reported for red deer in previous ecological studies, which may reflect species‐specific traits such as larger body size, stronger gregarious behavior, and less effective anti‐parasitic strategies compared to wild boar [13, 14, 65]. Nonetheless, a recent study identified wild boar as a suitable species for modeling CCHFV exposure risk [18]. Although not identified as a key determinant of local seroprevalence patterns in our analysis, this species may still contribute significantly to virus maintenance, particularly in anthropogenically modified landscapes such as hunting areas [70]. Artificial management practices such as supplementary feeding and water provisioning promote close spatial overlap between wild ungulates, which support high burdens of ticks. These common sources and shared habitats may serve as focal points for tick aggregation and virus transmission [65]. Hence, wild boar, in synergy with red deer, may sustain a silent enzootic tick–host–tick cycle.

Across the 18 study sites, our model identified red deer abundance as a significant positive predictor of CCHFV exposure, suggesting once again that landscapes dominated by this species are more likely to sustain viral circulation [17]. In contrast, higher relative abundance of small ruminants was linked to lower exposure levels. While sheep (*Ovis aries* Linnaeus, 1758) and goats (*Capra hircus* Linnaeus, 1758) act as hosts for both the virus and *Hyalomma* ticks [28, 30], several factors may reduce their epidemiological contribution. *Hyalomma* spp. exhibit a stronger preference for cattle, horses, and wildlife hosts, resulting in only sporadic parasitism of small ruminants in many Mediterranean systems, which is consistent with the generally low CCHFV seroprevalence reported in these species [20, 21, 71]. Their limited suitability for sustaining large tick populations likely plays a primary role, while the variable and often incomplete effects of antiparasitic treatments, such as avermectins, may further contribute to reducing viral amplification [72–74]. More importantly, areas dominated by small ruminants are typically pastoral systems with lower densities of wild ungulates, which can indirectly limit CCHFV circulation by constraining the distribution of *Hyalomma* spp. [32].

Historically, small ruminants shaped Mediterranean landscapes by maintaining open habitats and controlling shrub encroachment. The decline of these traditional pastoral systems, driven by rural exodus and a shift toward cattle‐based production [75, 76], has reduced grazing pressure and promoted shrub growth and woodland recovery across much of the Iberian Peninsula [77, 78]. Over the past two decades, forest cover has increased by ~ approximately 30% in Spain [79], creating more favorable conditions for wild ungulates such as red deer, which benefit from enhanced refuge and forage [80]. Consistently, our model identified forest land cover as a positive predictor of CCHFV exposure, likely reflecting the ecological suitability of Mediterranean forests for *H. lusitanicum*, a key vector species thriving in warm, wooded habitats with abundant wild hosts [32]. Altogether, the decline of small ruminant populations, forest expansion, and red deer proliferation appear to reinforce ecological conditions that sustain tick populations and facilitate the maintenance of CCHFV in Mediterranean landscapes.

Last, our 18‐site model underscored the role of climate in shaping CCHFV exposure risk. PSeas emerged as a significant positive predictor, consistent with previous findings [18]. High rainfall seasonality appears to favor the ecology of *H. lusitanicum*, which thrives in warm, dry environments but requires soil moisture to avoid desiccation [32, 65]. These conditions are characteristic of south‐central Iberia, where *H. lusitanicum* is among the most prevalent tick species [14, 81]. Moreover, strong seasonal precipitation may influence host behavior, driving wild ungulates to congregate around waterholes and shaded areas during periods of intense heat and drought [82]. These conditions may create *Hyalomma* spp. hot spots that increase tick‐host contact rates and facilitate the transmission of CCHFV [65].

A different set of predictors emerged when the analysis was restricted to sites with evidence of previous exposure to the virus. In these endemic areas, mammalian diversity was negatively associated with the odds of wild boar and red deer being exposed to the virus. This finding suggests that the presence of more diverse host communities provides a buffer against viral transmission. Lagomorph abundance also showed a negative association with exposure. Although these species have historically been considered key hosts for immature *Hyalomma* [28], ticks and reported as epidemiologically important in CCHF outbreaks in other regions [27], they do not appear to play the same epidemiological role in the Iberian Peninsula Infestation rates of *H. lusitanicum* on wild rabbits can be very high in deer‐rich habitats but decline markedly in the absence of deer [83], suggesting that lagomorphs alone are insufficient to sustain viable tick populations. In contrast, evidence indicates that *H. lusitanicum* populations can be maintained in deer‐dominated communities without rabbit involvement [81, 84]. Consequently, increasing lagomorph populations alongside declining wild ungulate numbers may reduce vector abundance and limit opportunities for virus amplification. This interpretation is consistent with the low CCHFV exposure observed in lagomorphs across Iberian serosurveys [23–25]. Small ruminant abundance did not show significant effects in this restricted model, implying that their influence is more relevant when comparing endemic with nonendemic landscapes rather than within established transmission foci. In addition, LST was positively associated with CCHFV circulation level in endemic regions. This can be explained by the fact that higher temperatures accelerate development and increase survival of *Hyalomma* ticks across life stages, while also extending their seasonal activity, thereby enhancing opportunities for pathogen transmission [28, 85].

This study had some limitations. First, despite the study sites covering a wide range of ecological contexts, the number of sites analyzed is limited when compared to the spatial scale of the Iberian Peninsula. To compensate for the low number of study sites (*n* = 18), we modeled the odds of individual exposure to CCHFV at the individual level (*n* = 1461). For futures studies, it would be beneficial to include sites in regions with more reported human CCHF cases. Sampling distribution was uneven across sites, with significant variations in sample size, particularly in sites where both wild boar and red deer were sampled. However, in these sites, the higher seroprevalence likely reflects ecological conditions favoring *Hyalomma* spp. rather than methodological inflation due to red deer inclusion. Moreover, the method used is only weakly sensitive to small mammals like rodents; combining it with other techniques would improve host community characterization and biodiversity metrics [86]. Using relative abundance to represent species presence gives a simplified view of population dynamics because it does not provide accurate estimates of the number of individuals, whereas population density data is more accurate but demands more rigorous methods [87]. Finally, although red deer and wild boar exhibit high exposure to CCHFV and are ecologically important hosts involved in virus maintenance [17, 18], their role as true amplifying hosts remains unconfirmed. To date, no studies have demonstrated measurable viremia, virus isolation, or successful transmission of CCHFV to feeding ticks in naturally infected individuals of these species. Therefore, any reference to their potential involvement in amplification should be interpreted strictly in an ecological sense. Future experimental and field‐based investigations are required to determine their host competence and to clarify whether they can support viral amplification under natural conditions.

## 5. Conclusion

Our findings highlight the central role of host community composition and diversity in modulating virus circulation. Red deer act as key ecologically hosts in CCHFV maintenance, sustaining silent enzootic cycles, especially within fenced hunting estates where their populations are artificially maintained at high densities. Conversely, more diverse communities, where this species is not dominant, appear to mitigate transmission risk. However, ongoing land‐use changes—including the abandonment of traditional grazing practices and consequent forest expansion—are favoring wild ungulate expansion and *Hyalomma* tick proliferation, thereby increasing the ecological suitability for CCHFV persistence. To counter this trend, effective management strategies should prioritize maintaining balanced host communities, promoting mixed grazing systems, and controlling wild ungulate populations. These objectives require coordinated action under an integrated One Health framework, involving ecological, veterinary, and public health sectors, alongside local stakeholders such as landowners, farmers, and hunters. This information is also relevant for designing targeted strategies for vaccine development and application to control CCHFV. Overall, this work emphasizes the impact of socioecological transformations on host community structure and how these in turn modulate the risk of CCHFV circulation.

## Author Contributions

Conceptualization: Patrícia Xavier, Alberto Perelló, and Christian Gortázar. Methodology: Patrícia Xavier and Alberto Perelló. Software, data curation, writing – original draft, visualization: Patrícia Xavier. Validation, supervision: Christian Gortázar. Formal analysis: Patrícia Xavier, Alberto Perelló, Víctor Luque‐Castro, David Relimpio, and Patricia Barroso. Investigation: Patrícia Xavier, Alberto Perelló, and David Relimpio. Resources: Patrícia Xavier, Ana Balseiro, and Christian Gortázar. Writing – review and editing: Patrícia Xavier, Alberto Perelló, Víctor Luque‐Castro, David Relimpio, Patricia Barroso, Ana Balseiro, Virgílio Almeida, José de la Fuente, Francisco Ruiz‐Fons, and Christian Gortázar. Project administration, funding acquisition: Christian Gortázar, Francisco Ruiz‐Fons, and Ana Balseiro.

## Funding

This work was supported by the Bio‐Graz Project (Grants PID2022‐141906OB‐C21 and PID2022‐141906OB‐C22), funded by MCIN/AEI/10.13039/501100011033 and FEDER, EU, and by the EcoEpi Project (Grant SBPLY/23/180225/000008), funded by the EU through the ERDF and by JCCM through INNOCAM. It was also supported by the Project 220418CONV, a management assignment agreement by which the Ministry of Agriculture, Fisheries and Food (MAPA) entrusted the University of Castilla‐La Mancha (UCLM) with tasks related to wildlife health management in Spain. Alberto Perelló and Víctor Luque‐Castro hold a predoctoral research contract at UCLM (Grant 2023‐UNIVERS‐11983), co‐funded by the European Social Fund Plus (ESF+).

## Ethics Statement

The present study was conducted using red deer and wild boar culled during regular hunting events or as part of official population control programs led by environmental authorities. No animals were killed specifically for the purposes of this research, and no specific ethical approval was required for sample collection.

## Consent

The authors have nothing to report.

## Conflicts of Interest

The authors declare no conflicts of interest.

## Supporting Information

Additional supporting information can be found online in the Supporting Information section.

## Supporting information


**Supporting Information 1** Figure S1 and Table S1. Figure S1 shows the spatial distribution of study points in the Iberian Peninsula. Table S1 lists the location and characteristics of study points, including bioregion, livestock presence, and sampled species.

**Supporting Information 2** Glossary of ecological indices with definitions and formulas for trapping rate (TR), relative occupancy index (ROI), relative weight (RW), relative interaction index (RII), and Shannon diversity index (*H*).

**Supporting Information 3** Table S2. Description and ecological relevance of environmental predictors used in modeling CCHFV exposure, including land surface temperature (LST), normalized difference vegetation index (NDVI), and precipitation seasonality (PSeas).

**Supporting Information 4** Table S3. Mean relative weight (RW) and standard deviation (SD) of host species groups across sites with high (≥50%) and null (0%) CCHFV seroprevalence, based on species’ potential role in CCHF epidemiology, with individual analysis for red deer, wild boar, lagomorphs, cattle, small ruminants, and other species grouped together.

**Supporting Information 5** Table S4. Relative weight of each species recorded through camera trapping at each study site, detailing community composition at each location.

**Supporting Information 6** Figures S2 and S3. Marginal effects of predictors on the probability of CCHFV exposure in wild boar and red deer, shown across 18 study points (Figure S2) and across 11 positive study points (Figure S3).

## Data Availability

The data that support the findings of this study are available from the corresponding author upon reasonable request.
