# Supplementary material for: Host Community Traits Driving Crimean‐Congo Hemorrhagic Fever Virus Maintenance in Iberian Ecosystems
Source: Transbound Emerg Dis. 2026 Mar 3;2026:1152849. doi: 10.1155/tbed/1152849 (PMC12954466; doi:10.1155/tbed/1152849)
Supplement: Supplementary file 1 — Supporting Information 1 Figure S1 and Table S1. Figure S1 shows the spatial distribution of study points in the Iberian Peninsula. Table S1 lists the location and characteristics of study points, including bioregion, livestock presence, and sampled species. [file TBED-2026-1152849-s003.docx]

**Supplementary material 1: Materials and methods**

Additional information on study design, sampling locations, and environmental context.

**
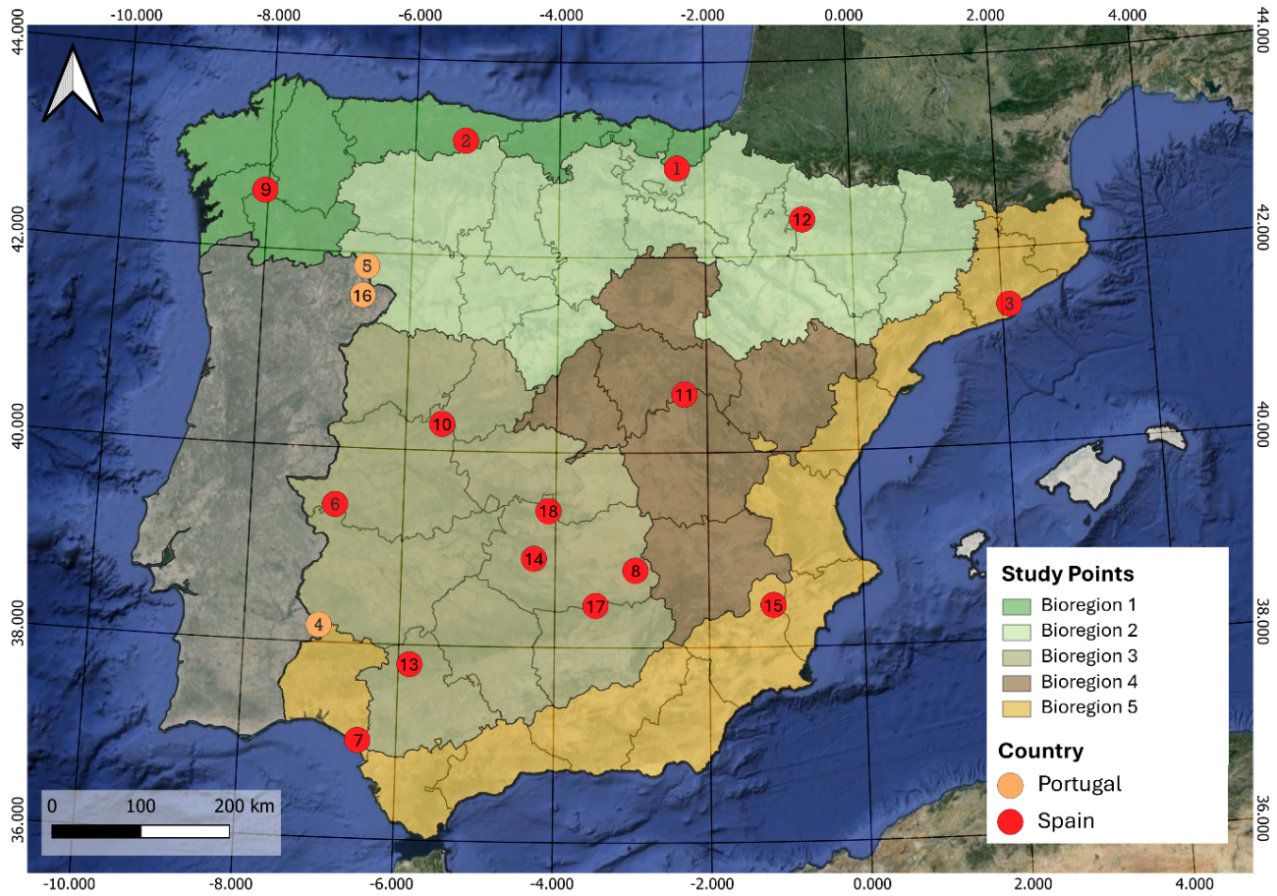
Figure S1.** Spatial distribution of study points in the Iberian Peninsula.

**Table S1.** Location and characteristics of study points.

| Study Point | Location | Bioregion | Type of Area | Presence of Livestock | Species Sampled |
| --- | --- | --- | --- | --- | --- |
| 1 | Álava | 2 | Open | Yes | Wb |
| 2 | Astúrias | 1 | Open | Yes | Wb |
| 3 | Barcelona | 5 | Open | No | Wb |
| 4 | Beja | 3 | Open | Yes | Wb |
| 5 | Bragança | 2 | Open | Yes | Wb |
| 6 | Cáceres | 3 | Fenced | No | Wb |
| 7 | Huelva | 5 | Open | Yes | Wb and Rd |
| 8 | Ciudad Real | 3 | Open | No | Wb |
| 9 | Pontevedra | 1 | Open | Yes | Wb |
| 10 | Ávila | 3 | Open | Yes | Wb and Rd |
| 11 | Guadalajara | 4 | Open | Yes | Wb and Rd |
| 12 | Huesca | 2 | Open | Yes | Wb and Rd |
| 13 | Sevilla | 3 | Fenced | No | Wb |
| 14 | Ciudad Real | 3 | Fenced | No | Wb and Rd |
| 15 | Murcia | 5 | Open | No | Wb |
| 16 | Bragança | 2 | Open | Yes | Wb |
| 17 | Ciudad Real | 3 | Fenced | No | Wb and Rd |
| 18 | Toledo | 3 | Fenced | No | Wb and Rd |

Abbreviations: Wb = Wild boar; Rd = Red deer. Bioregions follow the classification system of the Spanish Wildlife Disease Surveillance Scheme (Ministerio de Agricultura Pesca y Alimentación Español 2024). Presence or absence of livestock was based on phototrapping data.
