# Supplementary material for: Host Community Traits Driving Crimean‐Congo Hemorrhagic Fever Virus Maintenance in Iberian Ecosystems
Source: Transbound Emerg Dis. 2026 Mar 3;2026:1152849. doi: 10.1155/tbed/1152849 (PMC12954466; doi:10.1155/tbed/1152849)
Supplement: Supplementary file 2 — Supporting Information 2 Glossary of ecological indices with definitions and formulas for trapping rate (TR), relative occupancy index (ROI), relative weight (RW), relative interaction index (RII), and Shannon diversity index (H). [file TBED-2026-1152849-s005.docx]

**Supplementary material 2: Materials and methods**

Glossary of terms

1. Trapping rate (TR): The rate at which a target species is photographed by camera traps in a given area. Expressed as the number of independent detection events per number of camera trap days. From 0 to ∞.

$$TR=\frac{No. of records of target species}{Trapping effort of each study point}$$

1. Relative occupancy index (ROI): A simplified measure representing the proportion of surveyed sites where a target species is detected, used as a proxy for its spatial distribution. From 0 to 1.

$$ROI= \frac{No. of camera traps with records of target species}{Total number of camera traps}$$

2. Relative weight (RW): A comparative measure representing the relative contribution of a target species within a community. From 0 to 1.

$$RW= \frac{{(TR\times ROI)}_{target species}}{\sum_{i=1}^{n} {(TR\times ROI)}_{i}}$$

Where n is the total number of species

1. Relative interaction index (RII): A metric that quantifies the frequency of ecological interactions between two species. From 0 to ∞.

$$RII= \frac{No. of indirect interactions between two target species}{Trapping effort of each study point}$$

2. Shannon diversity index (H): A measure of species diversity of a community that combines both richness (the number of species) and evenness (the distribution of individuals among species). From 0 to ∞.

$$H= -\sum_{i=1}^{S} {(p}_{i}{ln p}_{i})$$

$p_{i}$ is the proportion of species *i* relative to the total number of species in the community (richness)
