# Supplementary material for: Host Community Traits Driving Crimean‐Congo Hemorrhagic Fever Virus Maintenance in Iberian Ecosystems
Source: Transbound Emerg Dis. 2026 Mar 3;2026:1152849. doi: 10.1155/tbed/1152849 (PMC12954466; doi:10.1155/tbed/1152849)
Supplement: Supplementary file 3 — Supporting Information 3 Table S2. Description and ecological relevance of environmental predictors used in modeling CCHFV exposure, including land surface temperature (LST), normalized difference vegetation index (NDVI), and precipitation seasonality (PSeas). [file TBED-2026-1152849-s006.docx]

**Supplementary material 3: Materials and methods**

| **Variable** | **Description** | **Ecological Relevance** |
| --- | --- | --- |
| **LST** (Land Surface Temperature) | Temperature of the ground | Influences tick development, survival, and activity. |
| **NDVI** (Normalized Difference Vegetation Index) | Greenness or density of vegetation | Indicates habitat suitability for ticks and their hosts, especially wild ungulates. |
| **PSeas** (Precipitation Seasonality) | Intra-annual variability of rainfall | Shapes water availability patterns that affect tick survival and host aggregation. |

**Table S2.** Description and ecological relevance of environmental predictors used in modelling the odds of CCHFV exposure.
