# Supplementary material for: Host Community Traits Driving Crimean‐Congo Hemorrhagic Fever Virus Maintenance in Iberian Ecosystems
Source: Transbound Emerg Dis. 2026 Mar 3;2026:1152849. doi: 10.1155/tbed/1152849 (PMC12954466; doi:10.1155/tbed/1152849)
Supplement: Supplementary file 4 — Supporting Information 4 Table S3. Mean relative weight (RW) and standard deviation (SD) of host species groups across sites with high (≥50%) and null (0%) CCHFV seroprevalence, based on species’ potential role in CCHF epidemiology, with individual analysis for red deer, wild boar, lagomorphs, cattle, small ruminants, and other species grouped together. [file TBED-2026-1152849-s004.pdf]

Supplementary material 4: Materials and methods

Table S3. Mean relative abundance (RW) and standard deviation (SD) of host species groups across sites with high (≥50%) and null (0%) CCHFV seroprevalence.

Host species	Seroprevalence	RW Mean (%)	RW SD (%)
**Red deer**<br>*Cervus elaphus* Linnaeus, 1758	High seroprevalence	69.6	11.0
	Seronegative	3.1	7.5
**Wild boar**<br>*Sus scrofa* Linnaeus, 1758	High seroprevalence	14.7	9.5
	Seronegative	36.8	26.9
**Lagomorphs**<br>Order *Lagomorpha*	High seroprevalence	4.1	2.2
	Seronegative	6.4	6.7
**Cattle**<br>*Bos taurus Linnaeus, 1758*	High seroprevalence	0.0	0.0
	Seronegative	1.8	2.3
**Small ruminants**<br>*Ovis aries Linnaeus, 1758*<br>*Capra hircus Linnaeus, 1758*	High seroprevalence	0.0	0.0
	Seronegative	0.8	1.1
**Other species**	High seroprevalence	11.6	12.7
	Seronegative	51.1	20.9
