# Supplementary material for: Host Community Traits Driving Crimean‐Congo Hemorrhagic Fever Virus Maintenance in Iberian Ecosystems
Source: Transbound Emerg Dis. 2026 Mar 3;2026:1152849. doi: 10.1155/tbed/1152849 (PMC12954466; doi:10.1155/tbed/1152849)
Supplement: Supplementary file 5 — Supporting Information 5 Table S4. Relative weight of each species recorded through camera trapping at each study site, detailing community composition at each location. [file TBED-2026-1152849-s002.pdf]

## Supplementary material 5: Materials and methods

**Table S4.** Relative weight of each species recorded through camera traps placed at each study point.

| | Study Point | | | | | | | | |
|---|---|---|---|---|---|---|---|---|---|
| **Species** | **1** | **2** | **3** | **4** | **5** | **6** | **7** | **8** | **9** |
| ***Sus scrofa* Linnaeus, 1758** | 41.01 | 10.63 | 84.08 | 16.25 | 6.13 | 5.22 | 38.95 | 12.83 | 20.01 |
| ***Cervus elaphus* Linnaeus, 1758** | 0 | 32.12 | 0 | 38.12 | 21.37 | 74.54 | 46.09 | 1.86 | 0 |
| ***Capreolus capreolus* (Linnaeus, 1758)** | 38.85 | 19.16 | 0.13 | 0 | 43.78 | 0.07 | 0.02 | 19.16 | 23.52 |
| ***Dama dama* (Linnaeus, 1758)** | 0.02 | 0 | 0 | 0 | 0 | 0.002 | 0 | 0 | 0 |
| ***Vulpes vulpes* (Linnaeus, 1758)** | 8.95 | 10.76 | 9.5 | 7.81 | 15.42 | 4.68 | 5.64 | 7.92 | 41.85 |
| **Order *Lagomorpha*** | 0.05 | 0.36 | 1.3 | 17.33 | 6.66 | 3.37 | 6.51 | 74.36 | 4.74 |
| ***Erinaceus europaeus Linnaeus, 1758*** | 0 | 0.13 | 0 | 0.05 | 0 | 0 | 0 | 0 | 0.1 |
| ***Martes* spp.** | 0.91 | 3.36 | 3.03 | 3.22 | 1.75 | 0.74 | 0 | 0.45 | 0.007 |
| ***Genetta genetta (Linnaeus, 1758)*** | 0.04 | 0.03 | 0.56 | 5.27 | 0 | 0.01 | 0.08 | 0.07 | 0.55 |
| ***Meles meles (Linnaeus, 1758)*** | 2.9 | 1.73 | 0.32 | 0.93 | 0.7 | 0.49 | 0.41 | 0.02 | 0.1 |
| ***Ovis gmelini musimon (Pallas, 1811)*** | 0 | 0 | 0 | 0 | 0 | 0.07 | 0 | 0 | 0 |
| ***Herpestes ichneumon (Linnaeus, 1758)*** | 0 | 0 | 0 | 0.2 | 0 | 0.03 | 0.18 | 0 | 0 |
| ***Ammotragus lervia (Pallas, 1777)*** | 0 | 0 | 0 | 0 | 0 | 0 | 0 | 0 | 0 |
| ***Capra pyrenaica Schinz, 1838*** | 0 | 0 | 0 | 0 | 0 | 0 | 0 | 0 | 0 |
| ***Rupicapra rupicapra (Linnaeus, 1758)*** | 0 | 1.24 | 0 | 0 | 0 | 0 | 0 | 0 | 0 |
| **Order *Rodentia*** | 0.91 | 15.09 | 0.73 | 3.12 | 0.84 | 0.04 | 0.03 | 2.16 | 1.7 |
| ***Felis* spp.** | 0.74 | 1.54 | 3.03 | 0 | 0 | 0.002 | 0 | 0.13 | 0.44 |
| ***Lutra lutra Linnaeus, 1758*** | 0 | 0 | 0 | 0 | 0 | 0 | 0 | 0 | 0 |
| ***Canis lupus signatus Cabrera, 1907*** | 0.02 | 0.17 | 0 | 0 | 0.14 | 0 | 0 | 0 | 0.01 |
| ***Ursus arctos Linnaeus, 1758*** | 0 | 0.003 | 0 | 0 | 0 | 0 | 0 | 0 | 0 |
| ***Lynx pardinus (Temminck, 1827)*** | 0 | 0 | 0 | 0 | 0 | 0 | 0.002 | 0 | 0 |
| ***Procyon lotor (Linnaeus, 1758)*** | 0 | 0 | 0 | 0 | 0 | 0 | 0 | 0 | 0 |
| ***Bos taurus Linnaeus, 1758*** | 5.17 | 3.48 | 0 | 4.88 | 0 | 0 | 0.66 | 0 | 5.36 |
| ***Ovis aries Linnaeus, 1758*** <br> ***Capra hircus Linnaeus, 1758*** | 0.02 | 0.02 | 0.08 | 2.83 | 3.26 | 0 | 0 | 0.02 | 1.7 |
| ***Equus caballus Linnaeus, 1758*** | 0 | 0.18 | 0 | 0 | 0 | 0 | 1.4 | 0 | 0 |
| ***Sus scrofa domestica Linnaeus, 1758*** | 0 | 0 | 0 | 0 | 0 | 10.72 | 0 | 0 | 0 |

**Supplementary material 5: Materials and methods**

**Table S4 (cont).** Relative weight of each species recorded through camera traps placed at each study point.

| | Study Point | | | | | | | | |
|---|---|---|---|---|---|---|---|---|---|
| **Species** | **10** | **11** | **12** | **13** | **14** | **15** | **16** | **17** | **18** |
| ***Sus scrofa* Linnaeus, 1758** | 8.47 | 13.86 | 66.31 | 31.47 | 17.62 | 29.2 | 10.85 | 13 | 6.26 |
| ***Cervus elaphus* Linnaeus, 1758** | 50.35 | 25.71 | 0.13 | 62.43 | 55.59 | 0 | 0 | 71.12 | 84.17 |
| ***Capreolus capreolus* (Linnaeus, 1758)** | 20.03 | 32.33 | 9.19 | 0.05 | 0 | 0 | 54.93 | 0.05 | 0.01 |
| ***Dama dama* (Linnaeus, 1758)** | 0 | 8.74 | 0 | 0.001 | 0 | 0 | 0 | 0.75 | 0.01 |
| ***Vulpes vulpes* (Linnaeus, 1758)** | 12.92 | 4.02 | 12.99 | 4.8 | 8.46 | 41.67 | 23.66 | 5.24 | 3.88 |
| **Order *Lagomorpha*** | 0 | 9.17 | 4.26 | 0.98 | 5.09 | 20.3 | 7.82 | 6.86 | 4.22 |
| ***Erinaceus europaeus Linnaeus, 1758*** | 0 | 0 | 0 | 0 | 0 | 0 | 0 | 0 | 0 |
| ***Martes* spp.** | 4.11 | 2.9 | 1.34 | 0.163 | 0.04 | 1.44 | 0.42 | 0.36 | 0.18 |
| ***Genetta genetta (Linnaeus, 1758)*** | 0.002 | 0 | 0.02 | 0.069 | 0.02 | 0.42 | 0.28 | 0.12 | 0.09 |
| ***Meles meles (Linnaeus, 1758)*** | 0.69 | 0.84 | 1.9 | 0.03 | 0.02 | 0.2 | 0.14 | 0.19 | 0.05 |
| ***Ovis gmelini musimon (Pallas, 1811)*** | 0 | 0 | 0 | 0 | 10.75 | 0 | 0 | 0 | 0 |
| ***Herpestes ichneumon (Linnaeus, 1758)*** | 0.03 | 0 | 0 | 0.001 | 0.003 | 0 | 0 | 0.002 | 0 |
| ***Ammotragus lervia (Pallas, 1777)*** | 0 | 0 | 0 | 0 | 0.91 | 0.02 | 0 | 0 | 0 |
| ***Capra pyrenaica Schinz, 1838*** | 0.07 | 0.2 | 0.02 | 0 | 0 | 0.04 | 0 | 0 | 0 |
| ***Rupicapra rupicapra (Linnaeus, 1758)*** | 0 | 0 | 0 | 0 | 0 | 0 | 0 | 0 | 0 |
| **Order *Rodentia*** | 0.21 | 0.61 | 0 | 0 | 1.5 | 6.66 | 0 | 2.35 | 0.19 |
| ***Felis* spp.** | 0.11 | 0 | 0 | 0.004 | 0 | 0 | 0 | 0.005 | 0.01 |
| ***Lutra lutra Linnaeus, 1758*** | 0 | 0 | 0.05 | 0.001 | 0 | 0 | 0 | 0.002 | 0 |
| ***Canis lupus signatus Cabrera, 1907*** | 0 | 0 | 0 | 0 | 0 | 0 | 0 | 0 | 0 |
| ***Ursus arctos Linnaeus, 1758*** | 0 | 0 | 0 | 0 | 0 | 0 | 0 | 0 | 0 |
| ***Lynx pardinus (Temminck, 1827)*** | 0 | 0 | 0 | 0 | 0 | 0 | 0 | 0 | 0 |
| ***Procyon lotor (Linnaeus, 1758)*** | 0 | 0 | 0 | 0 | 0 | 0 | 0 | 0 | 0 |
| ***Bos taurus Linnaeus, 1758*** | 2.79 | 0.01 | 0 | 0 | 0 | 0 | 1.76 | 0 | 0 |
| ***Ovis aries Linnaeus, 1758 Capra hircus Linnaeus, 1758*** | 0 | 1.61 | 0 | 0 | 0 | 0.04 | 0.14 | 0 | 0 |
| ***Equus caballus Linnaeus, 1758*** | 0.23 | 0 | 0 | 0 | 0 | 0 | 0 | 0 | 0 |
| ***Sus scrofa domestica Linnaeus, 1758*** | 0 | 0 | 0 | 0 | 0 | 0 | 0 | 0 | 0 |
