# Supplementary material for: Host Community Traits Driving Crimean‐Congo Hemorrhagic Fever Virus Maintenance in Iberian Ecosystems
Source: Transbound Emerg Dis. 2026 Mar 3;2026:1152849. doi: 10.1155/tbed/1152849 (PMC12954466; doi:10.1155/tbed/1152849)
Supplement: Supplementary file 6 — Supporting Information 6 Figures S2 and S3. Marginal effects of predictors on the probability of CCHFV exposure in wild boar and red deer, shown across 18 study points (Figure S2) and across 11 positive study points (Figure S3). [file TBED-2026-1152849-s001.docx]

**Supplementary material 6: Results**

**Fig S2.** Marginal effects of each predictor on the probability of exposure to CCHFV in wild boar and red deer individuals (n = 1461) across 18 study points in the Iberian Peninsula, as estimated from the best fitting generalized linear mixed model (GLMM). Each panel depicts the influence of a distinct predictor: (a) trapping rate of red deer, (b) trapping rate of small ruminants, (c) relative interaction index between large wild and domestic ungulates, (d) precipitation seasonality, and (e) forest cover. Shaded areas represent 95% confidence interval.


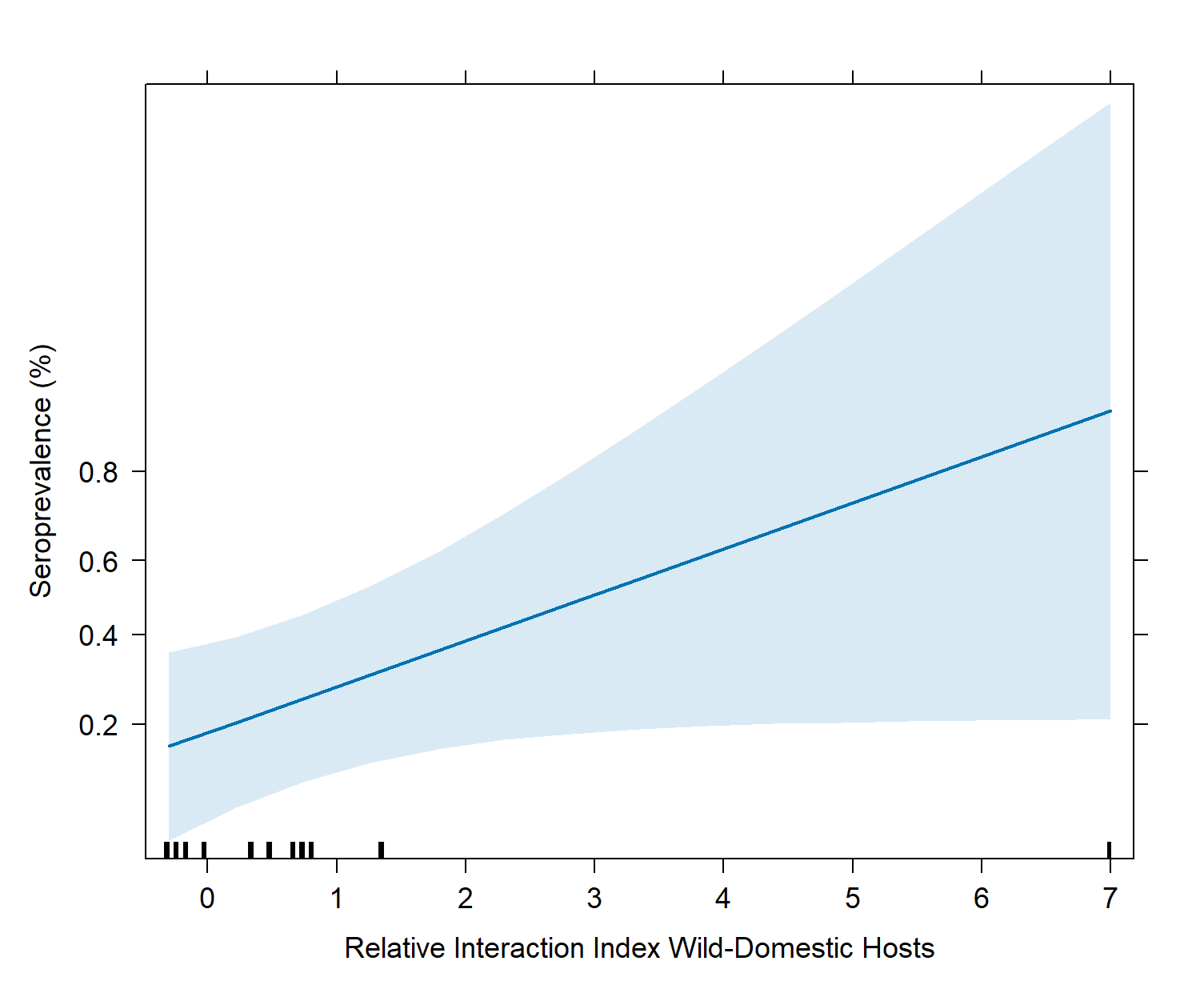

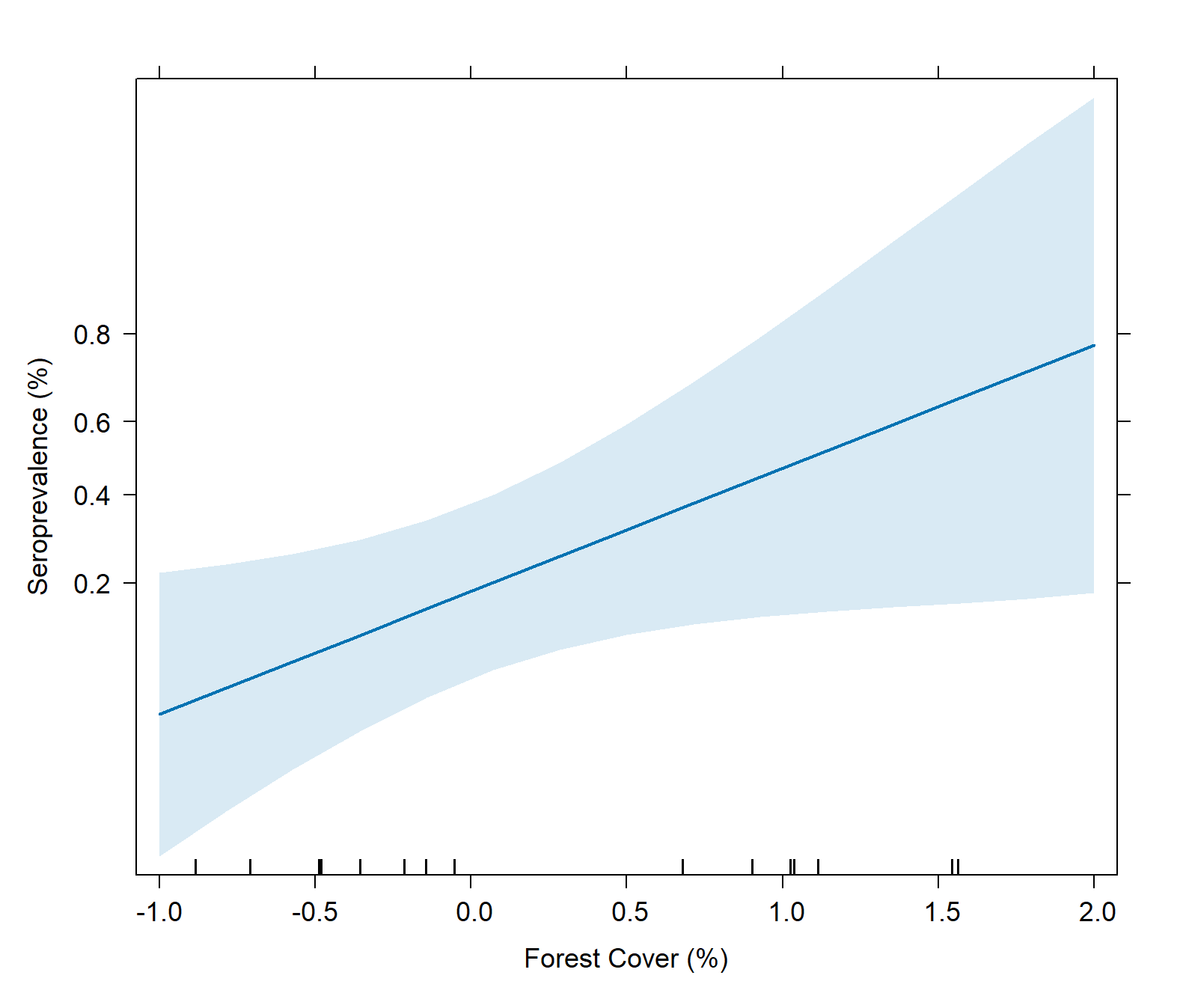

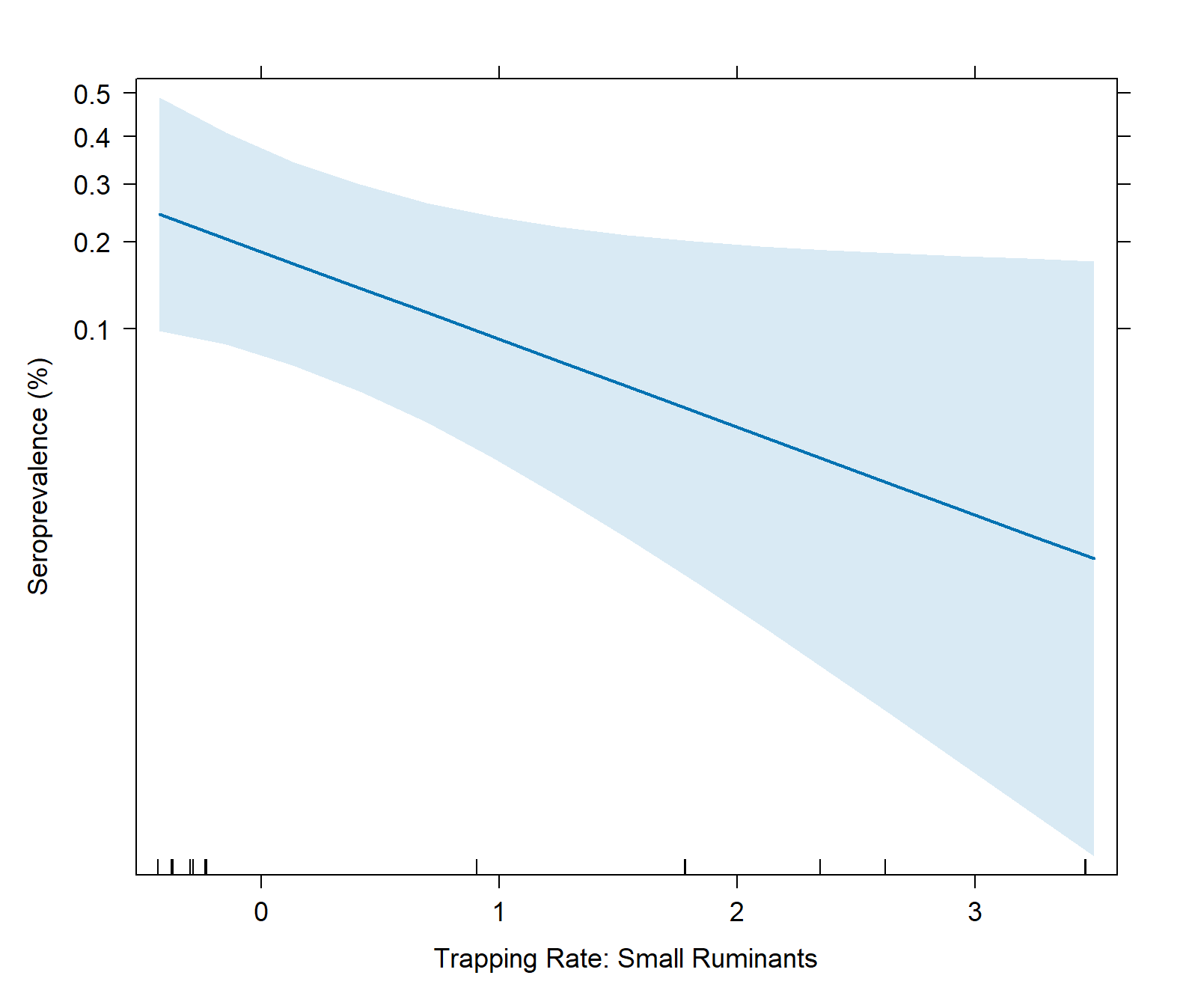

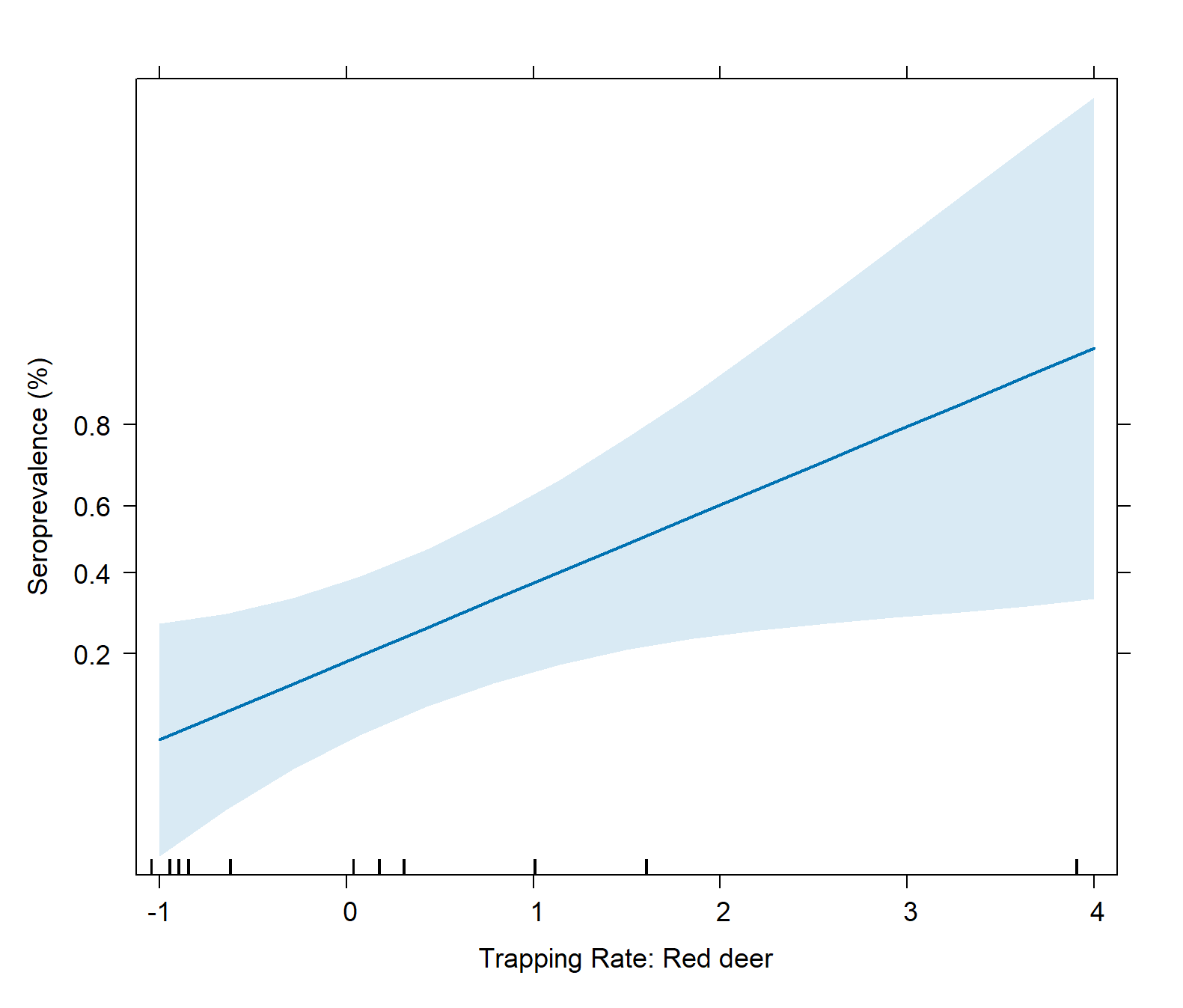

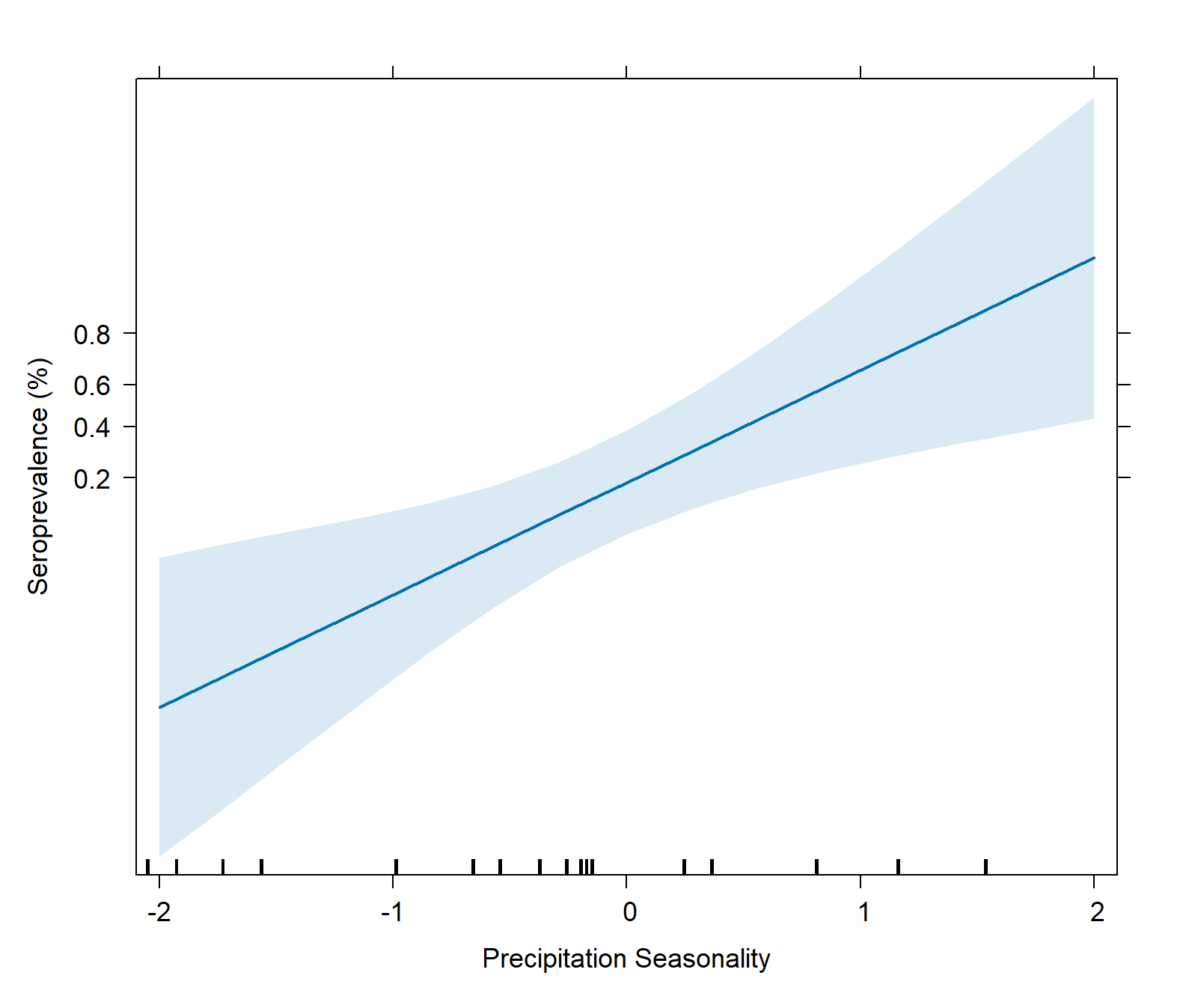


**a.**

**d.**

**b.**

**e.**

**c.**

**Fig S3.** Marginal effects of each predictor on the probability of exposure to CCHFV in wild boar and red deer individuals (n = 1157) across 11 positive study points in the Iberian Peninsula, as estimated from the best fitting generalized linear mixed model (GLMM). Each panel depicts the influence of a distinct predictor: (a) trapping rate for lagomorphs, (b) trapping rate for small ruminants, (c) Shannon diversity index of mammals, (d) mean annual land surface temperature, and (e) grass cover. Shaded areas represent 95% confidence interval.


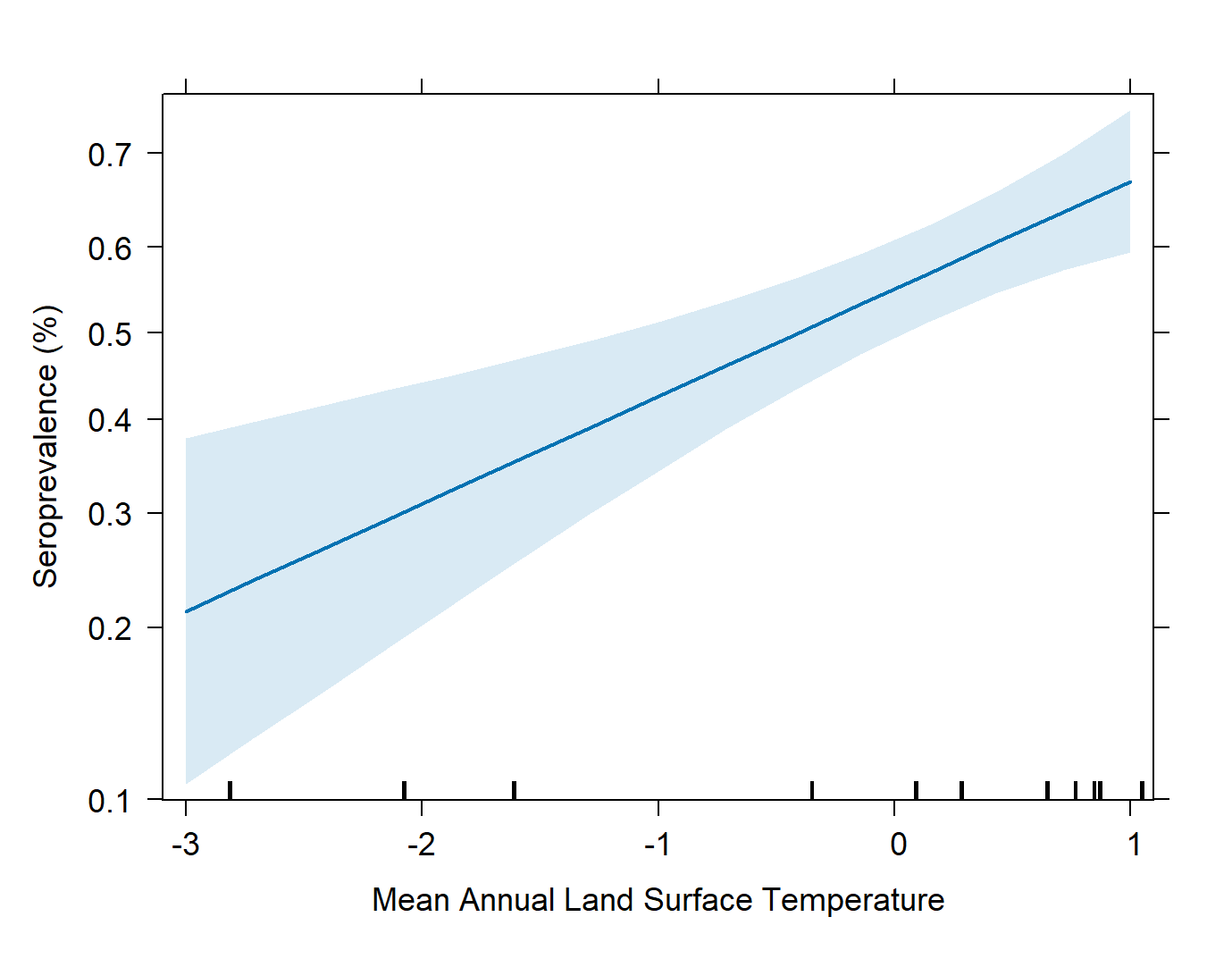

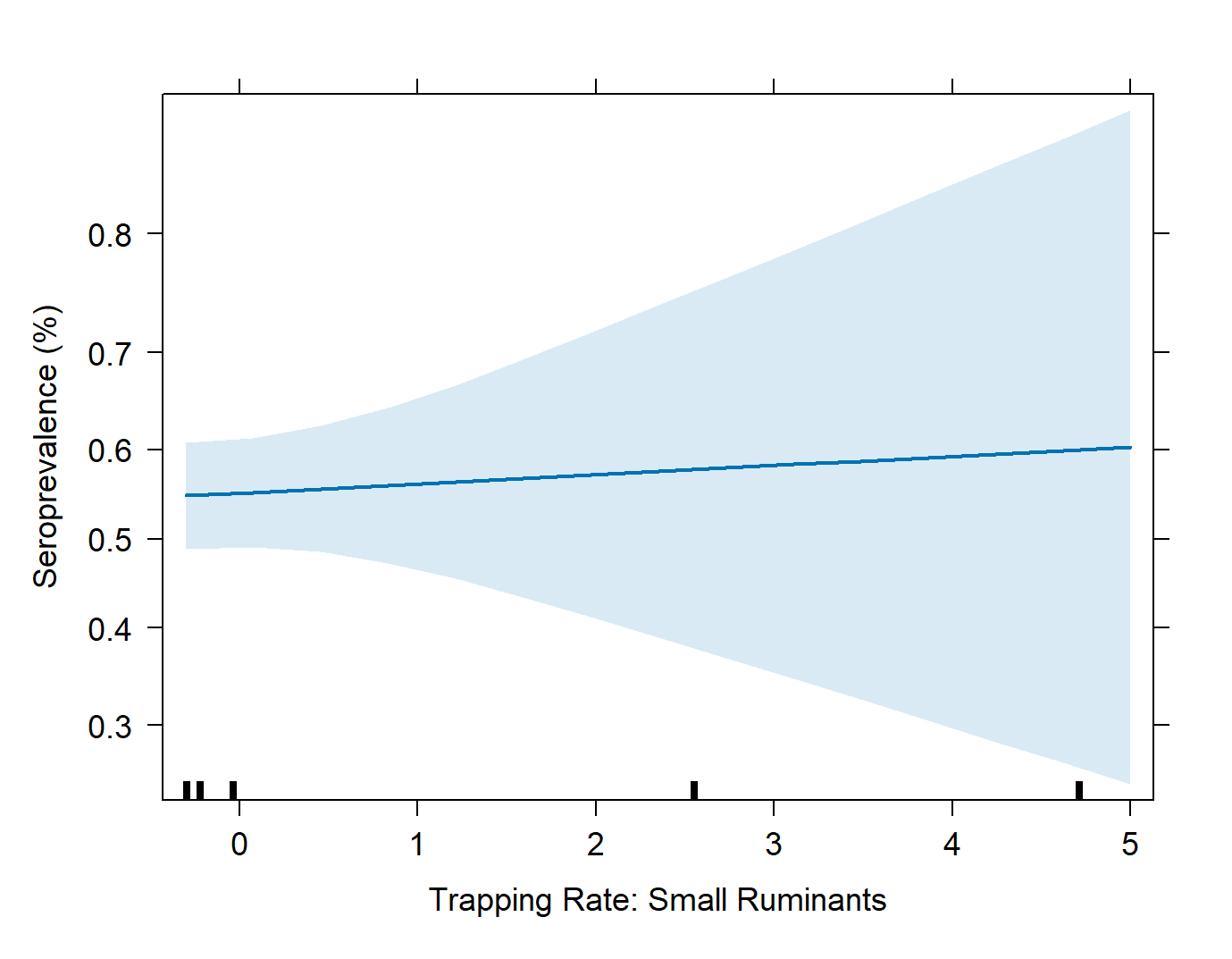

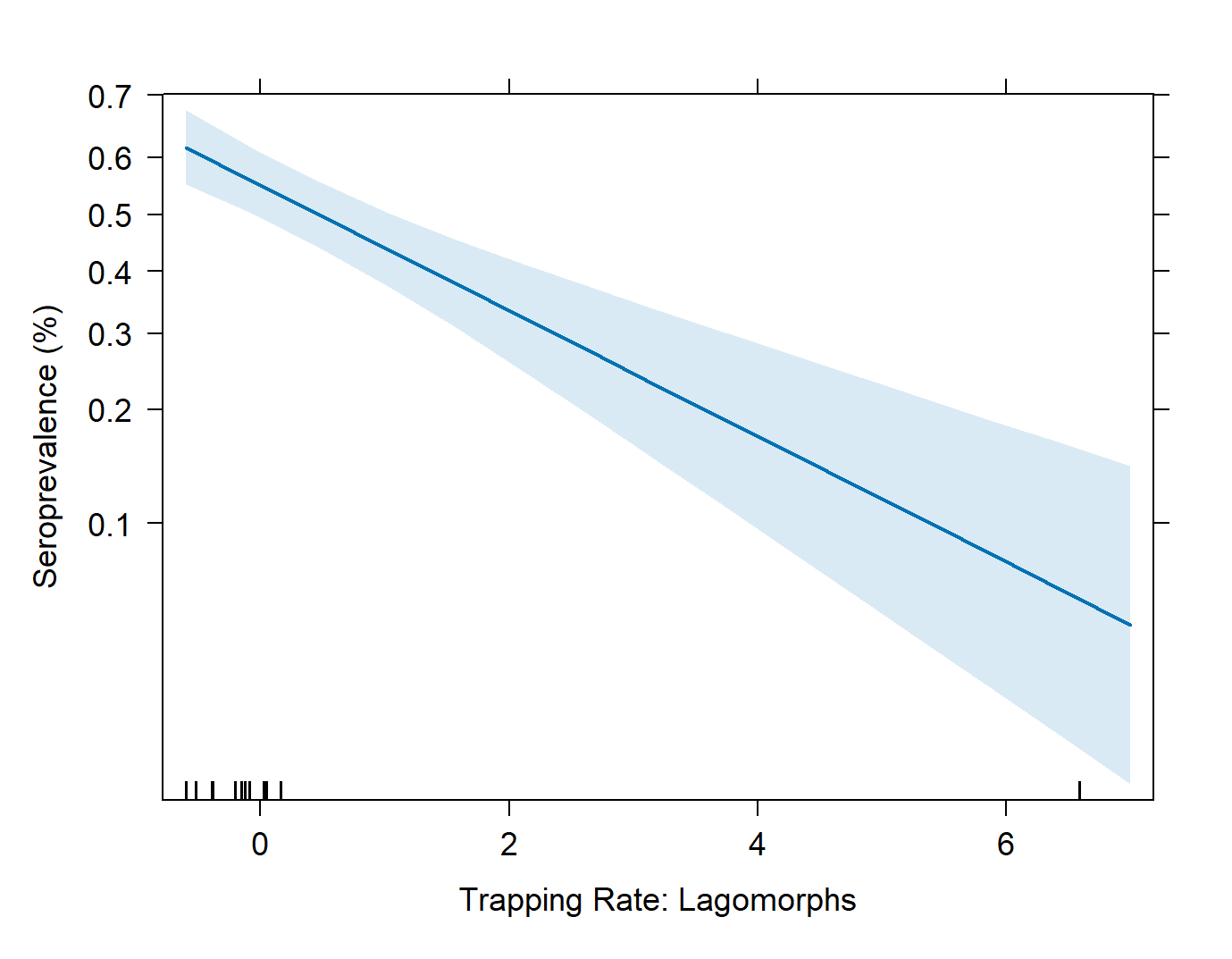

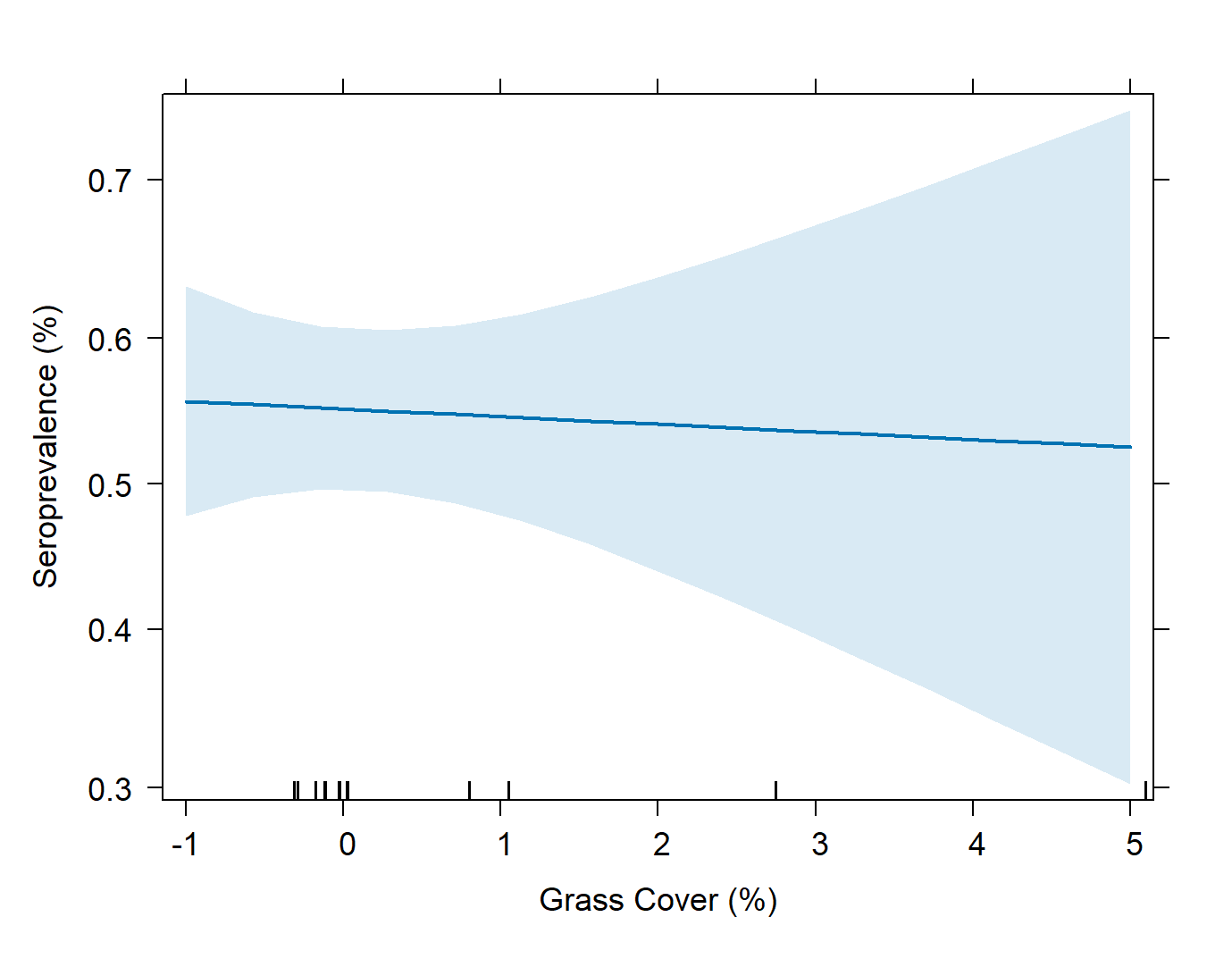


**a.**

**d.**

**b.**


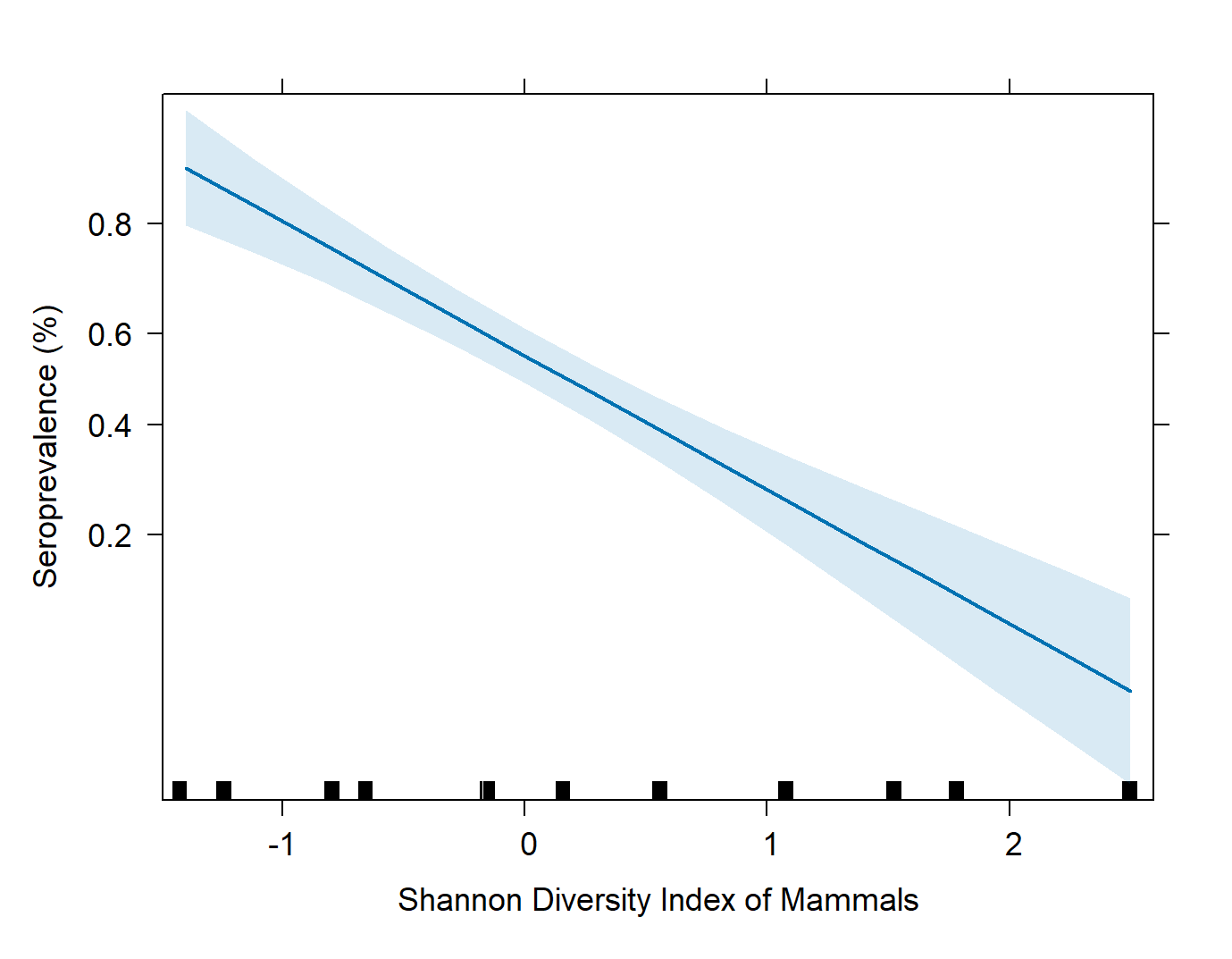


**e.**

**c.**
